# Association of endoscopic intervention with mortality in gastrointestinal bleeding after open-heart surgery: a propensity-score cohort study

**DOI:** 10.3389/fmed.2026.1713252

**Published:** 2026-04-22

**Authors:** Hailin Du, Yamei Sun, Guoyan Bai, Jie Zhang

**Affiliations:** 1 Department of Gastroenterology, Beijing Anzhen Hospital, Capital Medical University, Beijing, China; 2 Department of Gastroenterology, The Fifth Clinical Medical College of Henan University of Chinese Medicine (Zhengzhou People’s Hospital), Zhengzhou, Henan, China

**Keywords:** endoscopic intervention, gastrointestinal bleeding, mortality, open-heart surgery, propensity score full matching

## Abstract

**Introduction:**

Postoperative gastrointestinal bleeding (GIB) after open-heart surgery is associated with increased mortality, yet the prognostic impact of endoscopic intervention remains uncertain.

**Methods:**

We conducted a single-center retrospective cohort study of adults who developed GIB within 30 days after open-heart surgery (2017–2024). Patients were categorized into endoscopy and non-endoscopy groups, and propensity-score full matching was used to balance baseline covariates. The primary outcome was 30-day all-cause mortality; secondary outcomes were 1-year all-cause mortality, major postbleeding in-hospital complications (including acute myocardial infarction, acute respiratory distress syndrome, and other serious postoperative events), and postbleeding hospital length of stay.

**Results:**

Of 712 patients screened, 271 were included (endoscopy *n* = 68; non-endoscopy *n* = 203). After balancing baseline covariates, lower 30-day mortality was observed in the endoscopy group compared with the non-endoscopy group (adjusted mortality 26.5% vs. 49.8%; hazard ratio [HR] 0.54; 95% CI 0.32–0.91; *P* = 0.021, using a time-dependent Cox model). A similar pattern was observed at 1 year (HR 0.52; 95% CI 0.32–0.84; *P* = 0.007). No statistically significant difference was observed in major postbleeding in-hospital complications (odds ratio 1.86; 95% CI 0.94–3.68; *P* = 0.082, with residual uncertainty regarding potential harm) or prolonged length of stay (geometric mean ratio 1.17; 95% CI 0.99–1.39; *P* = 0.070). Endoscopy was associated with a higher cumulative incidence of discharge, accounting for death as a competing event (subdistribution HR 1.53; 95% CI 1.14–2.07; *P* = 0.005).

**Discussion:**

Among clinically eligible patients with postoperative GIB after open-heart surgery, lower 30-day and 1-year mortality rates were observed in those undergoing endoscopic intervention, whereas no statistically significant differences were observed in major postbleeding in-hospital complications or hospital length of stay. Given the retrospective nature of the study, these results should be considered hypothesis-generating rather than confirmatory, serving as a basis for future prospective investigations.

## Introduction

Gastrointestinal bleeding (GIB) is one of the most frequent GI complications after cardiac surgery and is associated with a substantial risk of mortality (1–4). While its overall incidence ranges from 0.2 to 2% (5), in a cohort of 6,316 coronary artery bypass grafting patients, those who developed postoperative GIB had a mortality rate of 47.6% (6), underscoring the clinical severity of this condition. Similarly, in a series of 2,056 open-heart surgery patients, gastrointestinal complications—over half of which were upper gastrointestinal bleeding—were associated with a 48.5% in-hospital mortality (7). In addition to high mortality, GIB significantly impairs clinical recovery; for instance, a study of 9,017 patients who underwent cardiac surgery showed that GIB more than doubled the length of hospital stay (23.5 ± 7.6 vs. 10.5 ± 7.1 days) (5).

As the cornerstone of GIB management, endoscopic intervention facilitates definitive diagnosis and hemostasis through modalities such as mechanical clipping, thermal coagulation, or application of topical agents (8); however, its application following open-heart surgery remains cautious and debated. To ensure optimal visualization and patient tolerance, most procedures are performed under moderate-to-deep sedation and, in select high-risk cases, under general anesthesia with endotracheal intubation (9). Clinicians, patients, and family members often hesitate due to concerns that sedation or anesthesia may exacerbate hemodynamic instability, potentially inducing malignant arrhythmias or respiratory failure in patients with severely limited cardiopulmonary reserve (10). Furthermore, endoscopy itself carries potential risks, including aspiration, mucosal injury, and, rarely, perforation (11). Consequently, conservative management is frequently favored (12).

Most existing research focuses on identifying risk factors for GIB rather than on assessing the clinical utility of endoscopic intervention (3, 4). Furthermore, available evidence is largely restricted to descriptive studies (5, 13). Crucially, long-term follow-up is lacking (14). Therefore, we conducted a propensity-score full-matched cohort study to evaluate the impact of endoscopic intervention on short-term (30-day) and long-term (1-year) all-cause mortality, major postbleeding in-hospital complications, and postbleeding hospital length of stay (LOS).

## Materials and methods

### Population

This retrospective single-center cohort study was performed at Beijing Anzhen Hospital, a tertiary cardiovascular center. Between January 2017 and January 2024, all consecutive adult patients with suspected gastrointestinal bleeding (GIB) within 30 days after cardiac surgery were initially screened for eligibility. GIB was defined as hematemesis, melena, or hematochezia accompanied by a hemoglobin decrease of > 20 g/L (3, 15). The exclusion criteria were: (1) non-open-heart procedures; (2) occult bleeding with a decrease in hemoglobin of ≤ 20 g/L; (3) GIB > 30 days postoperatively; and (4) missing data for the key covariates for analysis.

### Intervention and comparator

Patients were categorized into the endoscopy group or non-endoscopy group by whether they underwent endoscopic intervention. The decision to perform endoscopy was made jointly by the gastroenterology, cardiac surgery, and anesthesiology teams in consultation with the patients and their family members. The intervention of interest was diagnostic and/or therapeutic endoscopy performed after the onset of GIB. All procedures were performed by gastroenterologists with experience performing more than 10,000 endoscopic procedures, in collaboration with cardiac surgeons and anesthesiologists, and in accordance with the British Society of Gastroenterology guidelines on sedation in gastrointestinal endoscopy (9) and the European Society of Gastrointestinal Endoscopy guideline on the diagnosis and management of non-variceal upper gastrointestinal hemorrhage (16).

Patients who did not undergo endoscopy received conservative management in accordance with the European Society of Gastrointestinal Endoscopy guidelines (16). Conservative therapy comprised high-dose proton pump inhibitor infusion, reversal of antithrombotic therapy when indicated, hemostatic resuscitation, adjunctive pharmacologic agents (e.g., octreotide), and advanced hemodynamic monitoring.

All patients, regardless of treatment group, received standardized perioperative and postoperative care, including median sternotomy under general anesthesia, systemic heparinization with protamine reversal as indicated, intensive care unit monitoring, and transfer to the general ward upon clinical stabilization.

#### Outcomes

##### Primary outcome

The primary outcome was 30-day all-cause mortality after GIB. Clinical data for each patient—including vital signs data and treatment records—were obtained from electronic medical records. Deaths were confirmed via the national vital registration system.

Secondary outcomes were: (1) 1-year all-cause mortality; (2) major postbleeding in-hospital complications, encompassing the following new-onset events occurring after GIB diagnosis: intra-aortic balloon pump (IABP) or extracorporeal membrane oxygenation (ECMO) support, dialysis-requiring acute kidney injury, acute myocardial infarction, acute respiratory distress syndrome, pneumonia, tracheostomy, deep sternal wound infection, spinal cord ischemia/infarction, cerebrovascular accident (CVA), or new-onset cardiac arrhythmia; and (3) postbleeding hospital LOS, calculated from the date of GIB diagnosis to the date of discharge. For all outcomes, the follow-up period was initiated at the time of GIB diagnosis for both groups. In the endoscopy group, the interval between diagnosis and the procedure was accounted for as unexposed time within the time-dependent Cox model framework, ensuring that observation windows were defined with equal precision across groups. Follow-up for 1-year mortality was complete for all patients and verified through the national vital registration system, ensuring accurate and unbiased outcome ascertainment. Procedure-related complications were defined as clinically significant adverse events directly attributable to endoscopy, occurring during or within 24 h after the procedure, including perforation, bleeding requiring intervention, infections, or cardiopulmonary events. Events were identified from medical records, procedural reports, and ICU charts, and adjudicated by experienced physicians.

### Study design

We used full matching on the logit of the propensity score to control for selection bias and balance baseline covariates. Propensity scores were estimated using logistic regression. The potential for nonlinear relationships for key continuous variables (EuroSCORE II, GBS score, and hemoglobin) was formally assessed using restricted cubic splines, and no significant deviations from linearity were found (all P-nonlinearity > 0.05). Furthermore, clinical interactions were screened and found not to improve model balance; thus, covariates were maintained in their linear functional form to prioritize model parsimony and weight stability. The following prespecified covariates were included in the propensity-score model: age, sex, EuroSCORE II (17), Glasgow–Blatchford bleeding score (18), left ventricular ejection fraction ≤ 50%, hemorrhagic shock within 48 h before bleeding, prebleeding hemoglobin (latest measurement within 24 h prior to bleeding), prebleeding red blood cell transfusion, prebleeding advanced organ support (vasopressors, mechanical ventilation, IABP, ECMO, or continuous renal replacement therapy), prebleeding resternotomy, and surgical category (i.e., coronary artery bypass grafting, valve, or aortic/other complex procedures).

Full matching was performed using the R package “MatchIt” (version 4.5.2)^1^ with no caliper (method = “full,” distance = “logit”) (19). This method operates by partitioning all subjects into optimal subclasses with variable matching ratios, minimizing global weighted distance on the logit scale to ensure every individual is assigned to a subclass (20). Each subclass contained at least one treated and one control patient. Propensity score weights generated from the full matching procedure were used in all subsequent analyses. Weighted effective sample sizes were calculated as the square of the sum of weights divided by the sum of squared weights. The distribution of weights was inspected to assess the presence of extreme values and their potential impact on the robustness of the estimates. This method has been increasingly adopted in cardiovascular outcomes research, including recent applications in propensity score-matched studies of acute heart failure (21).

Covariate balance was assessed using weighted standardized mean differences, with SMD < 0.10 considered to represent optimal balance and < 0.20 acceptable balance (22). Propensity score distributions showed good overlap (Supplementary Figure 1), supporting the positivity assumption.

The study protocol was approved by the Institutional Review Board of Beijing Anzhen Hospital (approval No. 2025160x). Due to the retrospective nature of the analysis of existing data, the board waived the need for individual informed consent. The study was performed in accordance with the ethical principles of the Declaration of Helsinki and reported following the Strengthening the Reporting of Observational Studies in Epidemiology (STROBE) guidelines (23).

### Statistical analysis

For the unmatched cohort, categorical variables were summarized as counts (proportions) and compared using Pearson’s χ^2^ test or Fisher’s exact test, as appropriate. Continuous variables were summarized as medians with interquartile ranges (IQRs) and compared using the Wilcoxon rank-sum test. For the matched cohort, survey weights derived from full matching were applied. Categorical variables were compared using the Rao–Scott χ^2^ test, and continuous variables were compared using survey-weighted Wilcoxon tests (24).

The primary outcome, 30-day all-cause mortality, was analyzed using a time-dependent Cox proportional hazards model, in which endoscopy exposure was treated as a time-varying covariate. Patients were considered unexposed until the procedure and exposed thereafter. The secondary outcome of 1-year all-cause mortality was analyzed in the same way. Follow-up was administratively censored at 30 and 365 days, respectively. Effect sizes are reported as hazard ratios (HRs) with 95% confidence intervals (CI). The multivariable models were adjusted for age, sex, surgical category, shock within 48 h prior to bleeding, and prebleeding platelet count (latest measurement within 24 h prior to bleeding). The proportional hazards assumption for Cox models was verified using Schoenfeld residuals. To evaluate the stability of our findings and the risk of overfitting, the events-per-variable (EPV) ratio was assessed for all multivariable models, and the fragility index was calculated for the primary outcome.

Major in-hospital complications were analyzed using survey-weighted logistic regression analysis adjusted for prespecified covariates; results are expressed as odds ratios (ORs) with 95% CI. Individual complications were compared using Rao–Scott chi-square tests, and *P*-values were corrected for multiple testing using the Benjamini–Hochberg false discovery rate method.

Postbleeding hospital LOS was modeled via survey-weighted log-linear regression as log (LOS + 1), with results expressed as geometric mean ratios (GMRs) with 95% CI. Time to discharge was evaluated using a Fine–Gray subdistribution hazards model, with in-hospital death treated as a competing risk; results are reported as subdistribution hazard ratios (sHRs) with 95% CI. To estimate absolute risk differences and numbers needed to treat (NNT), we applied doubly robust estimation using Augmented Inverse Probability Weighting (AIPW). This method integrates a propensity score model and an outcome regression model to adjust for confounders. Standard errors and 95% CI were derived from 500 bootstrap iterations.

In the endoscopy group, we assessed initial hemostasis success and 7-day rebleeding rates. To explore how the intervention influenced survival, causes of death within 30 days were retrospectively adjudicated and categorized as bleeding-related, infection/multi-organ failure (MOF), cardiac-related, or other/unknown.

### Sensitivity analyses

To test the robustness of our primary findings, we performed five prespecified sensitivity analyses.

1. Weighted Cox regression.

A weighted Cox proportional hazards model incorporating the matching weights was performed for comparison with the primary time-dependent Cox results. Weighted Kaplan–Meier curves were plotted, and weighted log-rank tests were applied to provide a baseline comparison using traditional survival analysis methods.

2. Nearest-neighbor propensity score matching (1:2).

Patients who underwent endoscopy were propensity-score matched to non-endoscopy controls at an intended ratio of 1:2 (caliper 0.2 on the logit scale). Mortality was re-estimated in the matched cohort using a time-dependent Cox model, adjusted for the same covariates as in the primary analysis.

3. *E*-value calculation.

We computed the *E*-value for the fully adjusted hazard ratio from the primary analysis to quantify the minimum strength of association an unmeasured confounder would require, with both exposure and outcome, to nullify the observed effect. Calculations were performed with the *E*-Value package in R (25).

4. Doubly robust estimation.

Doubly robust estimation using AIPW, integrating a propensity-score model and an outcome regression model with the same covariates as the primary analysis, was performed to confirm the robustness of absolute risk estimates.

5. Adjustment for antithrombotic therapy.

To evaluate whether baseline antithrombotic use could have confounded the association between endoscopy and mortality, we fitted survey-weighted Cox models that included all covariates from the primary model, with further adjustment for antiplatelet therapy, high-intensity anticoagulation, or both.

All statistical analyses were performed using R (version 4.5.0; R Foundation for Statistical Computing, Vienna, Austria).

## Results

### Baseline characteristics and endoscopic features

**FIGURE 1:**
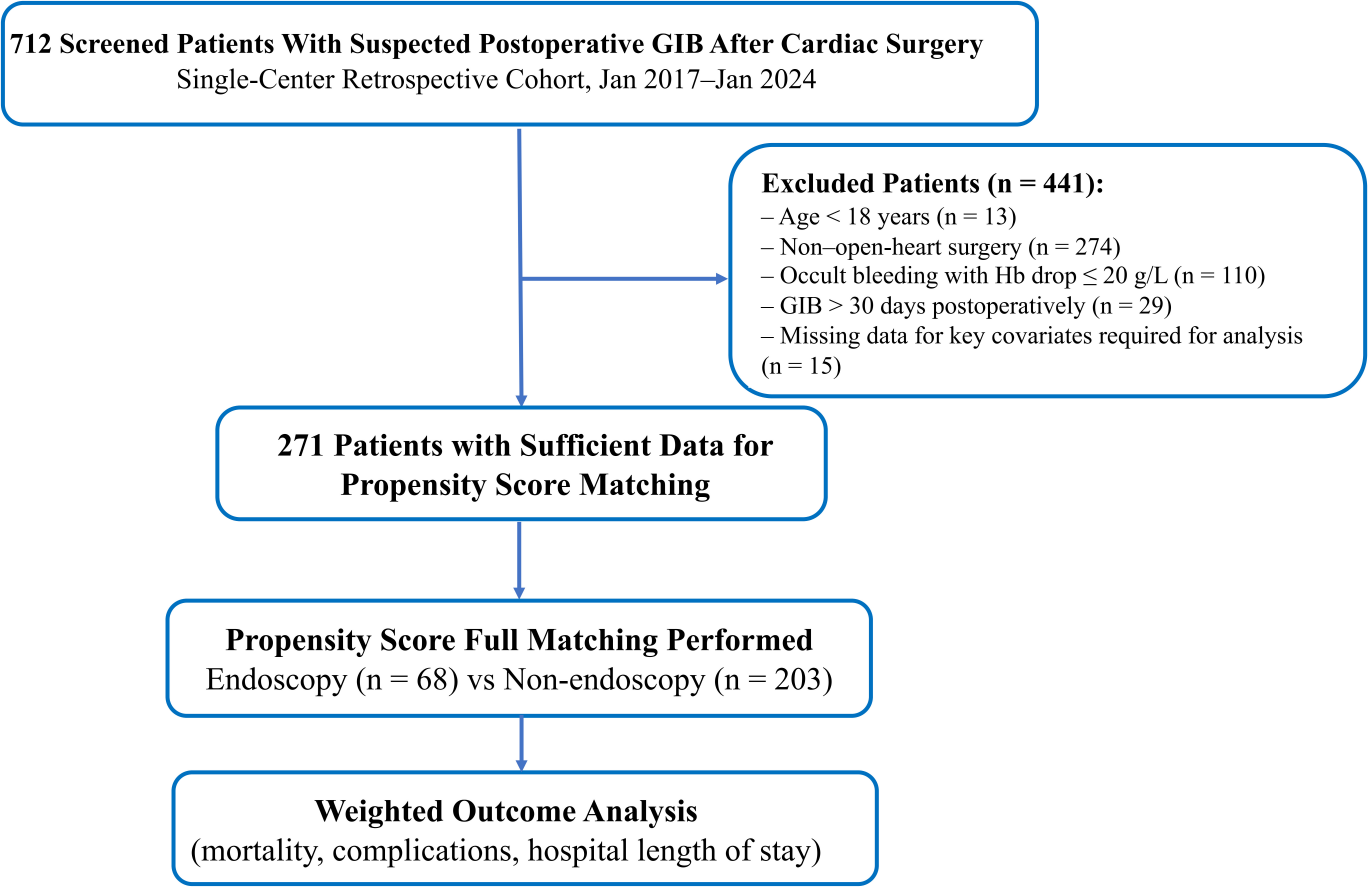
Study flowchart and propensity score full matching. Of 712 screened patients with suspected postoperative gastrointestinal bleeding (GIB) after cardiac surgery, 271 met all eligibility criteria and were included in the analysis. Full matching on the logit of the propensity score was performed using data from 68 patients who underwent endoscopy and 203 non-endoscopy controls; all 271 patients were retained after matching. Weighted analyses based on full-matching-derived survey weights were applied for all outcome models. GIB, gastrointestinal bleeding; Hb, hemoglobin.

Of the 712 screened patients with suspected postoperative GIB after cardiac surgery, 441 were excluded for various reasons, including 15 patients with missing values for key covariates required for the propensity-score model. As the proportion of missing data was small (2.1%), the impact on cohort representativeness was likely minimal. This resulted in a complete-case cohort of 271 patients for analysis. All variables required for the propensity score model were complete in the analyzed cohort. The patient flow, including reasons for exclusion, is shown in Figure 1. Follow-up for the 1-year mortality outcome was 100% complete for all patients. Following propensity-score full matching, all 271 patients remained in the matched analysis (68 endoscopy; 203 non-endoscopy).

After full matching, 59 subclasses were formed with variable treated/control ratios across subclasses. Weighted effective sample sizes (calculated as the square of the sum of weights divided by the sum of squared weights) were 68 for the endoscopy group and 61.3 for the non-endoscopy group. Weights ranged from 0.09 to 11.94; inspection showed no clustering of high-weight cases in specific outcome groups, confirming that results were not driven by extreme outliers.

Baseline characteristics for the study cohort are summarized in Table 1 and Supplementary Table 1. For the covariates included in the propensity-score model, the Love plot (Supplementary Figure 2) shows that all SMDs were below 0.20, with the majority below 0.10. An evaluation of all baseline characteristics in Supplementary Table 1 identified five variables with a moderate residual imbalance between 0.10 and 0.20 (preoperative creatinine, prebleeding anticoagulant therapy, valve surgery, prebleeding RBC transfusion, and Glasgow–Blatchford score). The prebleeding platelet count remained the primary residual imbalance (SMD = 0.313) and was thus included as a covariate in all multivariable models to adjust for potential residual confounding. These results demonstrate that the full matching procedure substantially reduced baseline imbalances among the matching covariates.

**TABLE 1:** Baseline characteristics before and after full matching using survey weights.

Variable	Unadjusted				Adjusted			
	Endoscopy (*n* = 68)	Non-endoscopy (*n* = 203)	*P*-value	SMD	Endoscopy	Non-endoscopy	*P*-value	SMD
I. Preoperative								
Age, years	63.00 (55.75–68.00%)	63.00 (52.50–68.50%)	0.971	< 0.001	62.50 (54.50–68.00%)	62.50 (51.75–69.17%)	0.714	0.063
Female	15 (22.1%)	68 (33.5%)	0.105	0.257	22.1%	24.0%	0.785	0.045
BMI, kg/m^2^	25.08 (23.16, 27.77%)	25.18 (22.77, 27.11%)	0.939	0.043	24.97 (23.04, 27.70%)	24.46 (23.24, 26.44%)	0.905	0.024
EuroSCORE II	8.62 (4.49–14.25%)	8.91 (5.07–15.06%)	0.881	0.073	8.62 (4.46, 14.25%)	10.15 (5.02, 17.14%)	0.758	0.053
LVEF ≤ 50%	14 (20.6%)	26 (12.8%)	0.171	0.209	20.6%	17.3%	0.608	0.084
Diabetes	18 (26.5%)	43 (21.2%)	0.462	0.124	26.5%	24.9%	0.840	0.036
Chronic lung disease	4 (5.9%)	9 (4.4%)	0.743	0.065	5.9%	4.6%	0.724	0.056
Preoperative creatinine, μmol/L	80.40 (69.00–98.33%)	77.60 (65.35–102.40%)	0.376	0.031	80.40 (68.10, 97.50%)	80.38 (69.40, 101.92%)	0.955	0.151
Prebleeding antiplatelet therapy	29 (42.6%)	99 (48.85%)	0.463	0.123	42.6%	43.8%	0.893	0.023
Prebleeding anticoagulant therapy	9 (13.2%)	22 (10.8%)	0.751	0.073	13.2%	8.5%	0.371	0.153
II. Intraoperative								
Procedure category								
CABG	28 (38.2%)	90 (44.3%)	0.982	0.124	38.2%	39.1%	0.925	0.018
Valve	9 (13.2%)	29 (14.3%)	0.989	0.030	13.2%	16.8%	0.884	0.100
Aortic + other	33 (48.5%)	84 (41.4%)	0.836	0.143	48.5%	44.1%	0.985	0.089
Cardiopulmonary bypass (CPB)	49 (72.1%)	155 (76.4%)	0.584	0.098	72.1%	73.6%	0.842	0.035
Cardiopulmonary bypass time, min	178.00 (132.00–224.00%)	192.00 (152.50–239.50%)	0.272	0.180	172.50 (129.75, 223.75%)	192.00 (155.00, 243.19%)	0.661	0.066
Aortic cross-clamp used	46 (67.6%)	133 (65.5%)	0.863	0.045	67.6%	64.8%	0.727	0.061
Aortic cross-clamp time, min	107.50 (86.50–142.75%)	116.00 (96.00–149.00%)	0.179	0.074	107.00 (85.00, 141.50%)	113.17 (90.06, 164.69%)	0.682	0.061
Hypothermia during surgery	47 (69.1%)	139 (68.5%)	1.000	0.014	69.1%	68.6%	0.953	0.010
Intraoperative TEE	20 (29.4%)	45 (22.2%)	0.295	0.165	29.4%	26.0%	0.692	0.076
Total surgery duration, h	6.00 (4.75–8.00%)	6.00 (5.00–8.00%)	0.582	0.087	6.00 (4.00, 8.00%)	6.00 (4.55, 8.00%)	0.847	0.043
III. Postoperative (pre-bleed)								
Shock before bleed	7 (10.3%)	31 (15.3%)	0.412	0.149	10.3%	10.4%	0.988	0.002
Resternotomy prior to bleeding	5 (7.4%)	42 (20.7%)	0.020	0.390	7.4%	9.3%	0.660	0.069
Advanced organ support ≥ 1	45 (66.2%)	169 (83.3%)	0.005	0.398	66.2%	65.1%	0.906	0.022
High-intensity anticoagulation	38 (55.9%)	116 (57.1%)	0.968	0.025	55.9%	57.1%	0.893	0.024
Prebleeding hemoglobin level, g/L	90.00 (81.00–110.00%)	86.00 (79.00–98.00%)	0.042	0.251	89.50 (80.75, 108.50%)	89.00 (78.60, 102.00%)	0.961	0.009
Prebleeding platelet count, × 10^9^/L	149.50 (73.75–252.80%)	105.00 (50.00–187.00%)	0.007	0.376	148.00 (70.00, 250.40%)	140.20 (81.18, 200.54%)	0.324	0.313
Prebleeding INR	1.29 (1.11–1.53%)	1.25 (1.12–1.57%)	0.784	0.023	1.28 (1.11, 1.52%)	1.31 (1.18, 1.62%)	0.470	0.006
Prebleeding RBC transfusion	39 (57.4%)	163 (80.3%)	< 0.001	0.508	57.4%	63.7%	0.464	0.129
IV. At-bleed presentation								
Glasgow–Blatchford score	10.00 (8.00–12.00%)	9.00 (7.00–11.00%)	0.005	0.419	10.00 (8.00, 12.00%)	11.00 (8.00, 13.00%)	0.571	0.108

Categorical variables are shown as n (%) before matching and survey-weighted % after matching; continuous variables are shown as median (IQR) throughout. All patients were retained after propensity score full matching (Endoscopy *n* = 68; Non-endoscopy *n* = 203). Post-matching estimates are derived from propensity score full-matching weights. As weighting produces non-integer denominators, absolute counts (n) are not applicable and are therefore omitted from the post-matching columns. CPB and aortic cross-clamp times were summarized only among patients who underwent the procedure (0 min excluded). Balance was assessed using the standardized mean difference (SMD), with SMD < 0.20 indicating acceptable balance. *P*-values are two-sided; post-matching comparisons used survey-design–based tests. Definitions. Prebleeding hemoglobin, prebleeding platelet count, and prebleeding INR refer to the latest measurements within 24 h prior to bleeding. High-intensity anticoagulation was defined as therapeutic-dose intravenous (IV) heparin or low-molecular-weight heparin (≥ 1 mg/kg) within 24 h before bleeding. Advanced organ support ≥ 1 includes vasopressors, mechanical ventilation, IABP, ECMO, or CRRT before bleeding. BMI, body mass index; CABG, coronary artery bypass grafting; CPB, cardiopulmonary bypass; CRRT, continuous renal replacement therapy; ECMO, extracorporeal membrane oxygenation; IABP, intra-aortic balloon pump; INR, international normalized ratio; IQR, interquartile range; LVEF, left ventricular ejection fraction; NA, not applicable; RBC, red blood cell; SMD, standardized mean difference; TEE, transoesophageal echocardiography.

Adherence to the proportional hazards assumption was confirmed for all Cox models (global Schoenfeld test, all *P* > 0.05). To ensure model parsimony and safeguard against overfitting, the EPV ratio was assessed and found to be 23.6, exceeding the recommended threshold of 10.

**FIGURE 2:**
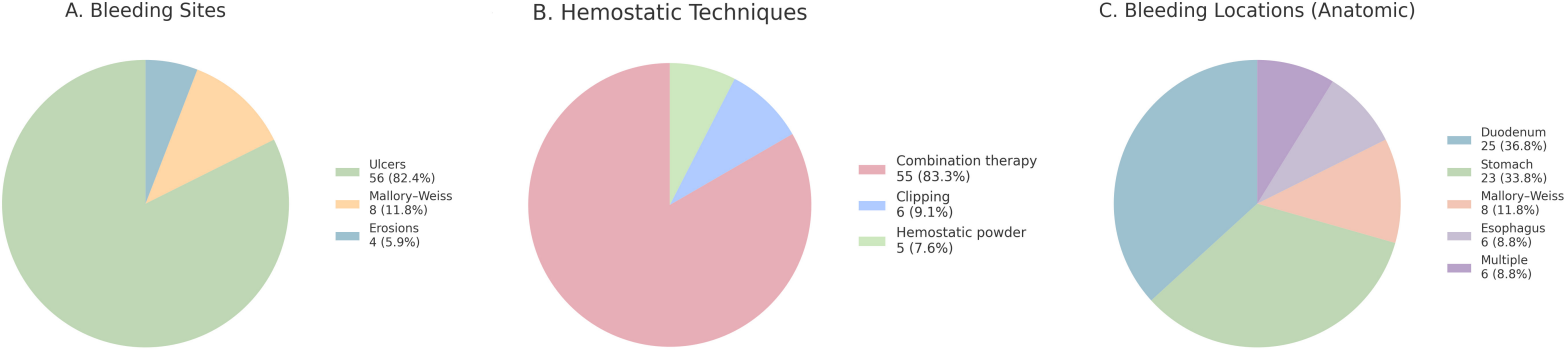
Endoscopic characteristics after gastrointestinal bleeding. **(A)** Bleeding etiologies among patients who underwent endoscopy (*n* = 68): peptic ulcer disease, *n* = 56 (82.4%), Mallory–Weiss tears, *n* = 8 (11.8%) and mucosal erosions, *n* = 4 (5.9%). **(B)** Hemostatic techniques among those receiving therapy (*n* = 66): combination therapy, *n* = 55 (83.3%), clipping, *n* = 6 (9.1%) and hemostatic powder, *n* = 5 (7.6%). **(C)** Anatomic bleeding locations among patients who underwent endoscopy (*n* = 68): duodenum, *n* = 25 (36.8%), stomach, *n* = 23 (33.8%), Mallory–Weiss region, *n* = 8 (11.8%), esophagus, *n* = 6 (8.8%) and multiple sites, *n* = 6 (8.8%). Initial hemostasis was achieved in 92.4% of the patients, with a 7-day rebleeding rate of 7.4%; no procedure-related complications were observed. Percentages are rounded to one decimal place and may not sum to 100%.

In the endoscopy group (*n* = 68), the most common bleeding etiology was peptic ulcer disease, followed by Mallory–Weiss tears and mucosal erosions (Figure 2A). Details of hemostatic techniques and anatomic bleeding locations are presented in Figures 2B,C. Initial hemostasis was achieved in 92.4% of cases, and the 7-day rebleeding rate was 7.4%, occurring at a median of 3 days post-procedure. Rebleeding was associated with a 30-day mortality rate of 80.0% (4/5), significantly higher than the 25.4% (16/63) observed in patients who achieved sustained hemostasis (*P* = 0.021; Supplementary Table 1). No procedure-related complications were reported.

### Primary outcomes

Lower 30-day all-cause mortality was observed in the endoscopy group compared with the non-endoscopy group. The primary time-dependent Cox model yielded an HR of 0.54 (95% CI: 0.32–0.91; *P* = 0.021) (Table 2). To provide marginal estimates of absolute effect, doubly robust estimation via AIPW was applied, showing an adjusted 30-day mortality of 26.5% vs. 49.8% (endoscopy versus non-endoscopy), representing an absolute risk reduction of 23.3% and an NNT of 4 (95% CI: 3–14) (Table 3). Raw, unadjusted event counts are also presented in Table 3 for transparency, with 18/68 (26.5%) deaths in the endoscopy group and 105/203 (51.7%) in the non-endoscopy group at 30 days.

**TABLE 2:** Association of endoscopic intervention with outcomes: survey-weighted models.

Model	Effect estimate	*P*-value
Time-dependent Cox model (30-day mortality)	HR = 0.54 (0.32–0.91)	0.021
Time-dependent Cox model (1-year mortality)	HR = 0.52 (0.32–0.84)	0.007
Logistic regression (major complications)	OR = 1.86 (0.94–3.68)	0.082
Log-linear model (postbleeding hospital LOS)	GMR = 1.17 (0.99–1.39)	0.070
Fine–Gray model (time to discharge; in-hospital death treated as a competing risk)	sHR = 1.53 (1.14–2.07)	0.005

Associations were estimated using survey-weighted regression: Time-dependent Cox model for 30-day and 1-year mortality; logistic regression for major in-hospital complications; log-linear regression of log(LOS + 1) with back-transformed geometric mean ratios (GMRs) for postbleeding length of stay; and Fine–Gray models for time to discharge (in-hospital death as a competing risk). All models incorporated weights from propensity score full matching with robust (sandwich) variance and were adjusted for age, sex, surgical category, prebleeding shock ( ≤ 48 h), and prebleeding platelet count. Values in parentheses are 95% confidence intervals. *P*-values are two-sided. HR, hazard ratio; OR, odds ratio; GMR, geometric mean ratio; sHR, subdistribution hazard ratio; LOS, length of stay; CI, confidence interval.

**TABLE 3:** Unadjusted and adjusted absolute risks for clinical events.

Outcome	Unadjusted mortality		Adjusted mortality		NNT (95% CI)
	Endoscopy (n, %)	Non-endoscopy (n, %)	Endoscopy (%)	Non-endoscopy (%)	
30-day mortality	18/68 (26.5%)	105/203 (51.7%)	26.5%	49.8%	4 (3–14)
1-year mortality	23/68 (33.8%)	127/203 (62.6%)	33.8%	57.1%	4 (3–13)
Major complications	28/68 (41.2%)	50/203 (24.6%)	41.2%	22.2%	-

Adjusted mortality was estimated using AIPW with full-matching-derived weights to account for baseline covariates. Raw, unadjusted event counts (%) are presented for transparency. The number needed to treat (NNT) was calculated as 1/| risk difference| and is reported with bootstrap-derived 95% confidence intervals. NNT was not calculated for major complications due to the heterogeneous nature of the composite endpoint. Relative effect estimates (Hazard Ratios or Odds Ratios) for these outcomes are provided in Table 2. CI, confidence interval; NNT, number needed to treat.

**FIGURE 3:**
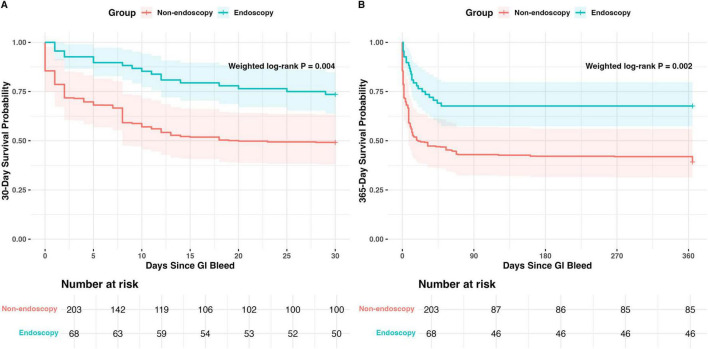
Weighted Kaplan–Meier survival curves for 30-day **(A)** and 365-day **(B)** all-cause mortality after gastrointestinal bleeding in the endoscopy and non-endoscopy groups. Shaded areas indicate 95% confidence intervals, and numbers at risk are shown below the curves. Weighted log-rank *P*-values were 0.004 for 30-day mortality and 0.002 for 365-day mortality.

Sensitivity analyses confirmed the robustness of these findings. Weighted Cox analysis incorporating full matching weights yielded an HR of 0.43 (95% CI: 0.24–0.76; *P* = 0.003), with weighted Kaplan–Meier curves showing clear separation between groups (Figure 3). Analyses using the 1:2 propensity-score-matched cohort and the full-matched cohort, both analyzed with time-dependent Cox models, produced effect estimates consistent in magnitude and direction with the primary analysis. The AIPW model produced consistent relative effect estimates, with risk ratios (RRs) of 0.53 (95% CI: 0.33–0.83) for 30-day mortality and 0.59 (95% CI: 0.39–0.84) for 1-year mortality. The fragility index for 30-day mortality was 11, and *E*-values were 4.08 at 30 days and 3.97 at 1 year (lower 95% CI limits: 1.96 and 2.12, respectively), indicating robustness to unmeasured confounding. In models further adjusted for antiplatelet therapy, high-intensity anticoagulation, or both—on top of all primary-model covariates—the HR for endoscopic intervention remained virtually unchanged from the primary analysis.

Exploratory analysis of cause-specific mortality showed consistently lower mortality rates across all categories in the endoscopy group, including lower incidences of bleeding-related deaths (7.4% vs. 12.8%), cardiac deaths (7.4% vs. 14.8%), and multi-organ failure (11.8% vs. 18.7%) (Supplementary Table 3).

### Secondary outcomes

One-year all-cause mortality was lower in the endoscopy group compared with the non-endoscopy group (HR, 0.52; 95% CI: 0.32–0.84; *P* = 0.007), estimated using the time-dependent Cox model (Table 2). Based on AIPW, the adjusted 1-year mortality was 33.8% vs. 57.1% (endoscopy versus non-endoscopy), representing an absolute risk reduction of 23.3% and an NNT of 4 (95% CI: 3–13) (Table 3). Raw, unadjusted event counts are also reported in Table 3: 23/68 (33.8%) in the endoscopy group and 127/203 (62.6%) in the non-endoscopy group.

No statistically significant differences were observed in the composite outcome of major postbleeding in-hospital complications following endoscopy (OR, 1.86; 95% CI: 0.94–3.68; *P* = 0.082; Table 2). The confidence interval is wide, reflecting uncertainty around the estimate. Procedural adverse events could not be separated from downstream hospital complications due to low incidence in this cohort. A detailed comparison of individual postoperative complications before and after matching is provided in Table 4. No single complication remained significant after Benjamini–Hochberg false discovery rate adjustment (all *q* > 0.05). While median postbleeding hospital LOS was 10.0 days (IQR: 6.0–17.0) in the endoscopy group and 6.0 days (IQR: 1.0–12.0) in the non-endoscopy group, adjusted analysis showed no statistically significant prolongation (geometric mean ratio, 1.17; 95% CI: 0.99–1.39; *P* = 0.070; Table 2). Using a Fine–Gray competing-risk model with death as a competing event, endoscopy was associated with a higher subdistribution hazard for discharge compared with non-endoscopy (subdistribution HR, 1.53; 95% CI: 1.14–2.07; *P* = 0.005; Table 2).

**TABLE 4:** Major postbleeding in-hospital complications after bleeding: unweighted counts and post-matching weighted %.

Variable	Unweighted			Post-matching (survey-weighted)		
	Endoscopy (*n* = 68)	Non-endoscopy (*n* = 203)	*P*-value	Endoscopy	Non-endoscopy	*P*-value
Any major postbleeding in-hospital complication	27 (39.7)	67 (33.0)	0.377	36.8%	25.4%	0.128
IABP or ECMO support	0 (0.0)	6 (3.0)	0.342	0.0%	1.9%	0.470
AKI requiring dialysis	10 (14.7)	13 (6.4)	0.061	8.8%	2.8%	0.092
Acute myocardial infarction (AMI)	0 (0.0)	1 (0.5)	1.000	0.0%	0.0%	0.574
Acute respiratory distress syndrome (ARDS)	3 (4.4)	2 (1.0)	0.103	2.9%	2.9%	1.000
Pneumonia	15 (22.1)	32 (15.8)	0.317	22.1%	11.9%	0.083
Tracheostomy	10 (14.7)	16 (7.9)	0.157	14.7%	3.9%	0.007
Deep sternal wound infection	3 (4.4)	8 (3.9)	1.000	4.4%	3.8%	0.848
Spinal cord infarction or ischemia	0 (0.0)	2 (1.0)	1.000	0.0%	0.1%	0.463
Cerebrovascular accident (CVA)	6 (8.8)	15 (7.4)	0.904	8.8%	3.5%	0.114
New-onset cardiac arrhythmia	5 (7.4)	13 (6.4)	0.781	7.4%	6.2%	0.761

Unweighted columns report n (%); post-matching columns report survey-weighted percentages derived from propensity score full matching, so absolute counts are not shown for post-matching estimates. Unweighted *P*-values were obtained using Pearson’s χ^2^ test (or Fisher’s exact test when expected counts were < 5); post-matching *P*-values were obtained using Rao–Scott χ^2^ tests from the survey design. All *P*-values are two-sided (α = 0.05). Multiple testing across individual complications was controlled using the Benjamini–Hochberg false-discovery-rate procedure (*q* < 0.05 regarded as significant). Detailed FDR-adjusted results are provided in Supplementary results. Major in-hospital complications include intra-aortic balloon pump (IABP) or extracorporeal membrane oxygenation (ECMO) support, dialysis-requiring acute kidney injury, postbleeding acute myocardial infarction (AMI), acute respiratory distress syndrome (ARDS), pneumonia, tracheostomy, deep sternal wound infection, spinal cord ischemia/infarction, cerebrovascular accident (CVA) and new-onset cardiac arrhythmia. IABP, intra-aortic balloon pump; ECMO, extracorporeal membrane oxygenation; AKI, acute kidney injury; FDR, false-discovery rate; q, FDR-adjusted *P*-value.

## Discussion

Among the 712 screened patients with suspected postoperative GIB after cardiac surgery during the study period, we analyzed data for 271 eligible cases, namely 68 who underwent endoscopic intervention and 203 managed non-endoscopically. After full matching using propensity scores, lower 30-day and 1-year mortality were observed among patients in the endoscopy group, while no statistically significant differences were noted in major postbleeding in-hospital complications or postbleeding hospital LOS compared with the non-endoscopy group. These results should be interpreted cautiously, as they are hypothesis-generating and may help guide future prospective studies.

The most common bleeding source identified in the endoscopy group was peptic ulcer, followed by Mallory–Weiss tears, with these two etiologies accounting for 94.2% of cases. In general upper GIB, peptic ulcers and Mallory–Weiss tears are common non-variceal causes, and endoscopic hemostasis is recommended as the standard therapeutic approach in clinical practice guidelines (26, 27). In our study of patients who underwent open-heart surgery, hemostasis was successfully achieved in 92.4% of cases, demonstrating that endoscopic hemostasis can be technically achieved in this high-risk population.

A study by Krawiec et al. (5) reported on 9,017 patients who underwent cardiac surgery. Among these patients, 91 (1.01%) underwent endoscopy for postoperative GIB, with duodenal ulcers being the source in 71% of cases. However, the prognostic implications of endoscopic intervention were not addressed in the study. In a series of 4,892 patients who underwent open-heart surgery (28), 18 (0.4%) developed GIB requiring endoscopic evaluation; duodenal ulcers accounted for 83% of cases. In patients who underwent cardiac surgery, GIB often results from multifactorial mucosal injury leading to peptic ulcers, potentially related to splanchnic hypoperfusion during prolonged cardiopulmonary bypass (29), systemic inflammation (30), and baseline gastrointestinal conditions (31). Intraoperative hypotension, vasopressor use, and postoperative opioid analgesia may further compromise mucosal defenses (28).

In our study of patients with GIB after open-heart surgery, the composite endpoint of major postbleeding in-hospital complications did not show statistically significant differences between groups; however, these results should be interpreted cautiously. The composite endpoint comprises clinically diverse events differing in severity, which may largely reflect patients’ baseline illness rather than procedural effects. The estimates remain imprecise, and the confidence intervals are compatible with both potential harm and no effect. No procedure-related adverse events were observed, although low-incidence events could not be assessed given the study size. Patients who underwent cardiac surgery often experience hemodynamic instability and have limited physiological reserves. For high-risk patients, endoscopy may be performed under anesthesiologist supervision, although evidence in postoperative cardiac populations remains limited (9). Similar findings have been reported in non-surgical high-risk cardiac populations. For example, a national analysis of over 1.2 million patients with acute myocardial infarction showed that endoscopy was not associated with increased procedural risk despite substantial comorbidity burden (32).

Regarding hospital stay, no significant difference in postbleeding hospital LOS was observed in our cohort between the endoscopy and non-endoscopy groups. A retrospective analysis by Okamoto et al. (13) of data for 1,625 patients who underwent cardiovascular surgery from 2011 to 2020 found that 47 patients (2.9%) underwent endoscopy for upper GIB within 30 days postoperatively. In the study, endoscopic intervention did not lead to an increase in LOS. This finding is consistent with our results. In Fine–Gray competing-risk models accounting for in-hospital death as a competing event, patients undergoing endoscopy showed a higher cumulative incidence of discharge.

Our study has limitations. First, the single-center retrospective design may limit external validity and potentially lead to an overestimation of the treatment effect magnitude. Although propensity-score full matching was used to reduce baseline imbalances, the possibility of residual selection bias and confounding by indication cannot be fully excluded. We adjusted for severity indicators at the time of bleeding (including shock and organ-support status) and performed multiple sensitivity analyses; nevertheless, residual confounding remains possible. Second, a minor imbalance in prebleeding platelet counts remained, though it was adjusted for in our models. Finally, our retrospective design cannot eliminate all sources of bias, but these findings provide preliminary evidence to support and guide future prospective, multicenter studies.

In conclusion, among selected patients deemed suitable for endoscopy following postoperative GIB, those who underwent endoscopic treatment were observed to have lower 30-day and 1-year mortality, while no statistically significant differences were seen in major postbleeding in-hospital complications or hospital length of stay compared with patients managed conservatively. Given the retrospective observational design, these results do not establish a causal effect of endoscopy on outcomes but rather highlight the clinical utility of the procedure in this setting. These findings should be considered hypothesis-generating and provide preliminary evidence to guide future prospective, multicenter studies.

## Data Availability

The raw data supporting the conclusions of this article will be made available by the authors upon reasonable request, subject to institutional and ethical requirements.

## Footnotes

1
www.r-project.org
