# Supplementary material for: Association of endoscopic intervention with mortality in gastrointestinal bleeding after open-heart surgery: a propensity-score cohort study
Source: Front Med (Lausanne). 2026 Apr 22;13:1713252. doi: 10.3389/fmed.2026.1713252 (PMC13143666; doi:10.3389/fmed.2026.1713252)
Supplement: Supplementary file 1 [file Data_Sheet_1.DOCX]

**Supplementary Figures and Tables**

Figure S1. Propensity score distributions before and after full matching.

Figure S2. Love plot of covariate balance before and after full matching.

Table S1. Standardized Mean Differences of Baseline Characteristics Pre- and Post-Full Matching.

Table S2. Impact of 7-day rebleeding on outcomes within the endoscopy group.

Table S3. Cause-specific mortality in the propensity-score full-matched cohort.


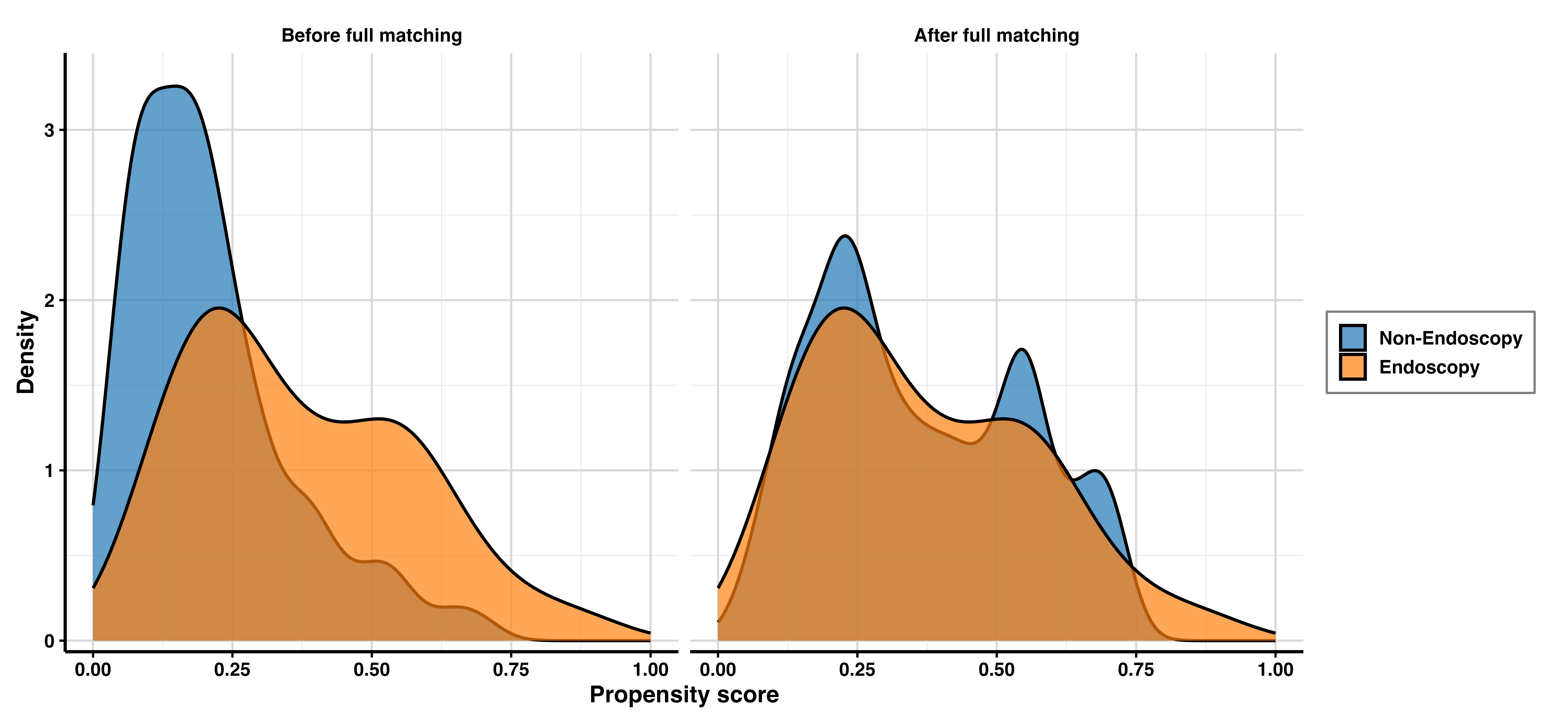


**Figure S1.** Density plots of propensity score distributions before (left) and after (right) full matching. Substantial overlap between the endoscopy and non-endoscopy groups supported the positivity assumption and indicated successful reduction of baseline confounding.

**
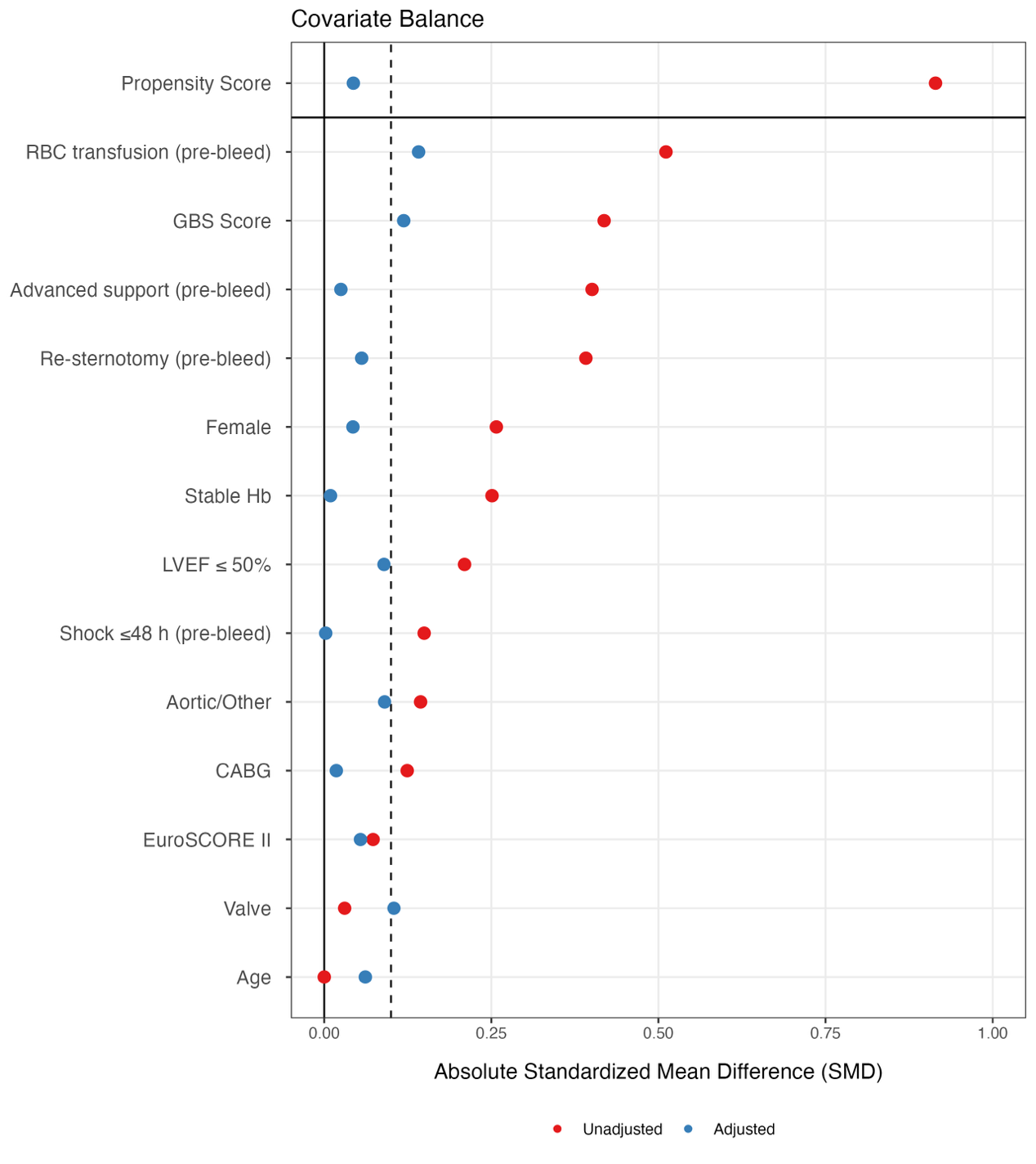
**

**Figure S2.** Love plot of covariate balance before and after full matching.

Standardized mean differences (SMDs) are shown for all matching covariates, with red points indicating the unadjusted sample and blue points the sample after full matching. Covariate balance improved substantially after matching; the dashed vertical line at |SMD| = 0.10 denotes the target threshold for optimal balance.

Abbreviations: GBS, Glasgow–Blatchford bleeding score; Hb, hemoglobin; RBC, red blood cell; pre-bleed, prebleeding; LVEF, left ventricular ejection fraction; CABG, coronary artery bypass grafting; EuroSCORE II, European System for Cardiac Operative Risk Evaluation II; Stable Hb, Prebleeding hemoglobin level.

**Table S1. Standardized Mean Differences of Baseline Characteristics Pre- and Post-Full Matching.**

| Variable | Pre-matching SMD | Post-matching SMD |
| --- | --- | --- |
| I. Preoperative | | |
| Age, years | <0.001 | 0.063 |
| Female | 0.257 ** | 0.045 |
| BMI, kg/m² | 0.043 | 0.024 |
| EuroSCORE II | 0.073 | 0.053 |
| LVEF ≤ 50% | 0.209** | 0.084 |
| Diabetes | 0.124* | 0.036 |
| Chronic lung disease | 0.065 | 0.056 |
| Preoperative creatinine, µmol/L | 0.031 | **0.151*** |
| Prebleeding antiplatelet therapy | 0.123* | 0.023 |
| Prebleeding anticoagulant therapy | 0.073 | **0.153*** |
| II. Intraoperative | | |
| Procedure category | | |
| CABG | 0.124* | 0.018 |
| Valve | 0.030 | **0.100*** |
| Aortic + other | 0.143* | 0.089 |
| Cardiopulmonary bypass (CPB) | 0.098 | 0.035 |
| Cardiopulmonary bypass time, min | 0.180* | 0.066 |
| Aortic cross-clamp used | 0.045 | 0.061 |
| Aortic cross-clamp time, min | 0.074 | 0.061 |
| Hypothermia during surgery | 0.014 | 0.010 |
| Intraoperative TEE | 0.165* | 0.076 |
| Total surgery duration, h | 0.087 | 0.043 |
| III. Postoperative (pre-bleed) | | |
| Shock before bleed | 0.149* | 0.002 |
| Resternotomy prior to bleeding | 0.390** | 0.069 |
| Advanced organ support ≥ 1 | 0.398** | 0.022 |
| High‑intensity anticoagulation | 0.025 | 0.024 |
| Prebleeding hemoglobin level, g/L | 0.251** | 0.009 |
| Prebleeding platelet count, ×10⁹/L | 0.376** | **0.313**** |
| Prebleeding INR | 0.023 | 0.006 |
| Prebleeding RBC transfusion | 0.508** | **0.129*** |
| IV. At‑bleed presentation | | |
| Glasgow–Blatchford score | 0.419** | **0.108*** |

**Notes:** Standardized mean differences (SMD) are reported to assess covariate balance between Non-Endoscopy and Endoscopy groups. Prior to matching, imbalances (SMD ≥ 0.20, marked with **) and moderate imbalances (SMD between 0.10 and 0.20, marked with *) were observed. Variables with residual post-matching SMD between 0.10 and 0.20 are indicated by **bolded numerical value***, while **bolded numerical value**** denotes post-matching SMD ≥ 0.20.

Table S2. Impact of 7-day rebleeding on outcomes within the endoscopy group.

| Variable | Patients  with Rebleeding  (n = 5) | Patients  without Rebleeding  (n = 63) | P value |
| --- | --- | --- | --- |
| 30-day Mortality, n (%) | 4 (80.0%) | 16 (25.4%) | 0.021 |
| 1-year Mortality, n (%) | 4 (80.0%) | 20 (31.7%) | 0.038 |

Note: P values were calculated using Fisher's exact test.

Table S3. Cause-specific mortality in the propensity score full-matched cohort.

| Cause of Death Classification | Endoscopy Group  (N = 68) | Non-Endoscopy Group (N = 203) |
| --- | --- | --- |
| Bleeding-related Mortality | 5 (7.4%) | 26 (12.8%) |
| Infection / Multi-organ Failure (MOF) | 8 (11.8%) | 38 (18.7%) |
| Cardiac-related Mortality | 5 (7.4%) | 30 (14.8%) |
| Other / Unknown | 2 (2.9%) | 28 (13.8%) |
| Total Deaths | 20 (29.4%) | 122 (60.1%) |

Note: Counts (n) and percentages (%) represent the observed data from the full cohort.
